# Frequency-Dependent Competition Between Strains Imparts Persistence to Perturbations in a Model of *Plasmodium falciparum* Malaria Transmission

**DOI:** 10.3389/fevo.2021.633263

**Published:** 2021-05-26

**Authors:** Qixin He, Shai Pilosof, Kathryn E. Tiedje, Karen P. Day, Mercedes Pascual

**Affiliations:** 1 Department of Biological Sciences, Purdue University, West Lafayette, IN, United States,; 2 Department of Life Sciences, Ben-Gurion University of the Negev, Beer-Sheva, Israel,; 3 Department of Microbiology and Immunology, Bio21 Institute, The University of Melbourne, Melbourne, VIC, Australia,; 4 Department of Ecology and Evolution, University of Chicago, Chicago, IL, United States,; 5 Santa Fe Institute, Santa Fe, NM, United States

**Keywords:** strain diversity, stabilizing competition, stochastic assembly, persistence, malaria and antigenic diversity, negative frequency-dependent selection, agent-based model, var genes

## Abstract

In high-transmission endemic regions, local populations of *Plasmodium falciparum* exhibit vast diversity of the *var* genes encoding its major surface antigen, with each parasite comprising multiple copies from this diverse gene pool. This strategy to evade the immune system through large combinatorial antigenic diversity is common to other hyperdiverse pathogens. It underlies a series of fundamental epidemiological characteristics, including large reservoirs of transmission from high prevalence of asymptomatics and long-lasting infections. Previous theory has shown that negative frequency-dependent selection (NFDS) mediated by the acquisition of specific immunity by hosts structures the diversity of *var* gene repertoires, or strains, in a pattern of limiting similarity that is both non-random and non-neutral. A combination of stochastic agent-based models and network analyses has enabled the development and testing of theory in these complex adaptive systems, where assembly of local parasite diversity occurs under frequency-dependent selection and large pools of variation. We show here the application of these approaches to theory comparing the response of the malaria transmission system to intervention when strain diversity is assembled under (competition-based) selection vs. a form of neutrality, where immunity depends only on the number but not the genetic identity of previous infections. The transmission system is considerably more persistent under NFDS, exhibiting a lower extinction probability despite comparable prevalence during intervention. We explain this pattern on the basis of the structure of strain diversity, in particular the more pronounced fraction of highly dissimilar parasites. For simulations that survive intervention, prevalence under specific immunity is lower than under neutrality, because the recovery of diversity is considerably slower than that of prevalence and decreased *var* gene diversity reduces parasite transmission. A Principal Component Analysis of network features describing parasite similarity reveals that despite lower overall diversity, NFDS is quickly restored after intervention constraining strain structure and maintaining patterns of limiting similarity important to parasite persistence. Given the described enhanced persistence under perturbation, intervention efforts will likely require longer times than the usual practice to eliminate *P. falciparum* populations. We discuss implications of our findings and potential analogies for ecological communities with non-neutral assembly processes involving frequency-dependence.

## 1. INTRODUCTION

Under negative frequency dependent selection (NFDS), the relative fitness of a species or a genotype decreases as its abundance and therefore its frequency increases. In the realm of population genetics, NFDS is a form of balancing selection promoting the coexistence of genotypes or of genes, with MHC being a prominent example (Aguilar et al., 2004; Key et al., 2014). In community ecology, NFDS causes species to limit themselves more than others, which stabilizes competition and maintains biodiversity by promoting species coexistence (HilleRisLambers et al., 2012). In some realms, such as microbial ecology, the boundary between these levels of organization is blurred, yet here too, NFDS has been shown to maintain microbial diversity (Cordero and Polz, 2014; Healey et al., 2016). Although the evolutionary time scales at which NFDS operates can be different for different organisms and levels of organization, the mechanisms underlying it can be common. In community ecology, competition for resources and selection for traits that give an edge in resource acquisition can shape the biodiversity of plants and animals (Browne and Karubian, 2016). For example, interaction with specific herbivores leads to negative density-dependent mortality and coexistence of congenerics *Inga spp* in forest ecosystems (Forrister et al., 2019). In infectious diseases, competition for hosts and selection for traits that allow pathogens to evade human immune systems shape the antigenic diversity of pathogens (Gupta and Day, 1994; Grenfell et al., 2004).

Beyond diversity *per se*, NFDS underlies the structure of such diversity in both pathogen populations (Koelle et al., 2006; Volz et al., 2013) and species communities (Scheffer and van Nes, 2006; D’Andrea et al., 2019). There is an unappreciated analogy between the structure of niches in trait space emerging from NFDS in these two lines of research (Pascual, 2020). One infectious disease system in which such strain structure has been investigated is that of the malaria parasite *Plasmodium falciparum*. Strain coexistence in local populations of the malaria parasite results from an on-going assembly process involving the dynamic interplay between ecology (population dynamics of strains, competition for hosts) and evolution (genetic changes via mutation and recombination) (He et al., 2018). The trait of interest concerns here the variation in the major antigen of the blood stage of infection PfEMP1. This protein is exported by the parasite to the surface of the infected blood cells where it becomes the target of the adaptive immune system (Bull et al., 1998). Variations of the protein, or variable surface antigens (VSAs), are encoded by a multigene family known as *var* with about fifty to sixty gene copies per parasite, whose expression is sequential and influences the duration of infection. From this perspective, an individual parasite corresponds to a unique combination of *var* genes—a “repertoire” —encoding for a particular set of VSAs. In high-transmission endemic regions, such as those of sub-Saharan Africa, local diversity of *var* gene types is in the order of tens of thousands. This extremely large genetic pool underlies the vast combinatorial diversity of the repertoires themselves. Both these high levels of diversity result from high recombination rates, which act at the two different levels of organization, mixing repertoires and generating new *var* genes, respectively (Larremore et al., 2013; Zilversmit et al., 2013).

Previous theory and data have shown that frequency-dependent competition for hosts is at play in determining the coexistence of a large number of repertoires, whose population structure is both non-random and non-neutral despite the vast gene pool (Barry et al., 2011; Day et al., 2017; He et al., 2018; Pilosof et al., 2019). Hosts can be viewed as resource patches whose availability depends on their individual history of “consumption” from previous exposure. This is because exposure to a specific VSA leads to the acquisition of specific immunity by individual hosts, which then precludes expression of the corresponding gene and thus shortens infection, reducing the fitness of the parasites that carry this gene. Adaptive immunity therefore implements selection, providing an advantage to strains carrying rare novel *var* genes, and a disadvantage to those composed of common ones. In the language of community ecology, the system experiences stabilizing competition (sensu Chesson, 2000) from trait variation that underlies niche differences. In that of population genetics, such competition is a mechanism of NFDS, which acts as a form of balancing selection and promotes strain (i.e., *var* repertoire) coexistence (He et al., 2018; Pilosof et al., 2019).

We have previously used the malaria system to address the challenge of discerning rules of assembly from complex population patterns, focusing on the large strain variation of *P. falciparum* populations in endemic regions of high transmission. We applied for this purpose a combination of stochastic agent-based models and network analyses of the genetic similarity between repertoires they generate (Artzy-Randrup et al., 2012; He et al., 2018; Pilosof et al., 2019). These studies have shown that networks assembled under NFDS differ in their topology from those assembled in the absence of selection, under neutral processes. Hence, in the malaria system the importance of individual and specific interactions can be detected in features of the macroscopic similarity structure of (strain) diversity. We note that networks, rather than phylogenetic trees, were applied because of the predominant role of recombination in the evolutionary change of the system.

In community ecology, the structure of diversity has been a major theoretical question, because of its hypothesized influence on system “stability” to perturbations (Hutchinson, 1959; Allesina and Tang, 2015). In infectious disease dynamics, a parallel yet little studied line of research regards the response of structured pathogen populations to interventions. Here, we illustrate the combination of stochastic ABMs and network analysis for this purpose, in *P. falciparum* populations under interventions that decrease transmission intensity. We ask whether the malaria system assembled under NFDS is more or less “stable” than a neutral counterpart. We apply press perturbations (Ives and Carpenter, 2007) in a stochastic computational model to represent interventions that target the mosquito vector with indoor residual spraying (IRS), which involves the application of an insecticide to internal walls and ceilings of homes (WHO, 2015). IRS effectively reduces transmission rate and therefore, the growth rate of the parasite population as a whole. We examine two components of stability: (i) persistence—the ability of the parasite’s population to withstand an intervention and (ii) recovery: the transient rebound of repertoire abundance or parasite prevalence, and of genetic diversity post-intervention. We consider in particular how quickly repertoire structure is restored. We end by drawing plausible analogies and implications for other ecological systems (Azarian et al., 2019; Forrister et al., 2019).

## 2. MATERIALS AND METHODS

### 2.1. Agent-Based Model (ABM) and Model Setup

Malaria transmission and intervention are modeled using an agent-based, discrete-event, continuous-time stochastic system described in detail in He et al. (2018) and Pilosof et al. (2019). Here, we briefly describe the agent-based model (ABM), with an emphasis on the specific implementation of the regional intervention scheme that constitutes the press perturbation.

**FIGURE 1 |:**
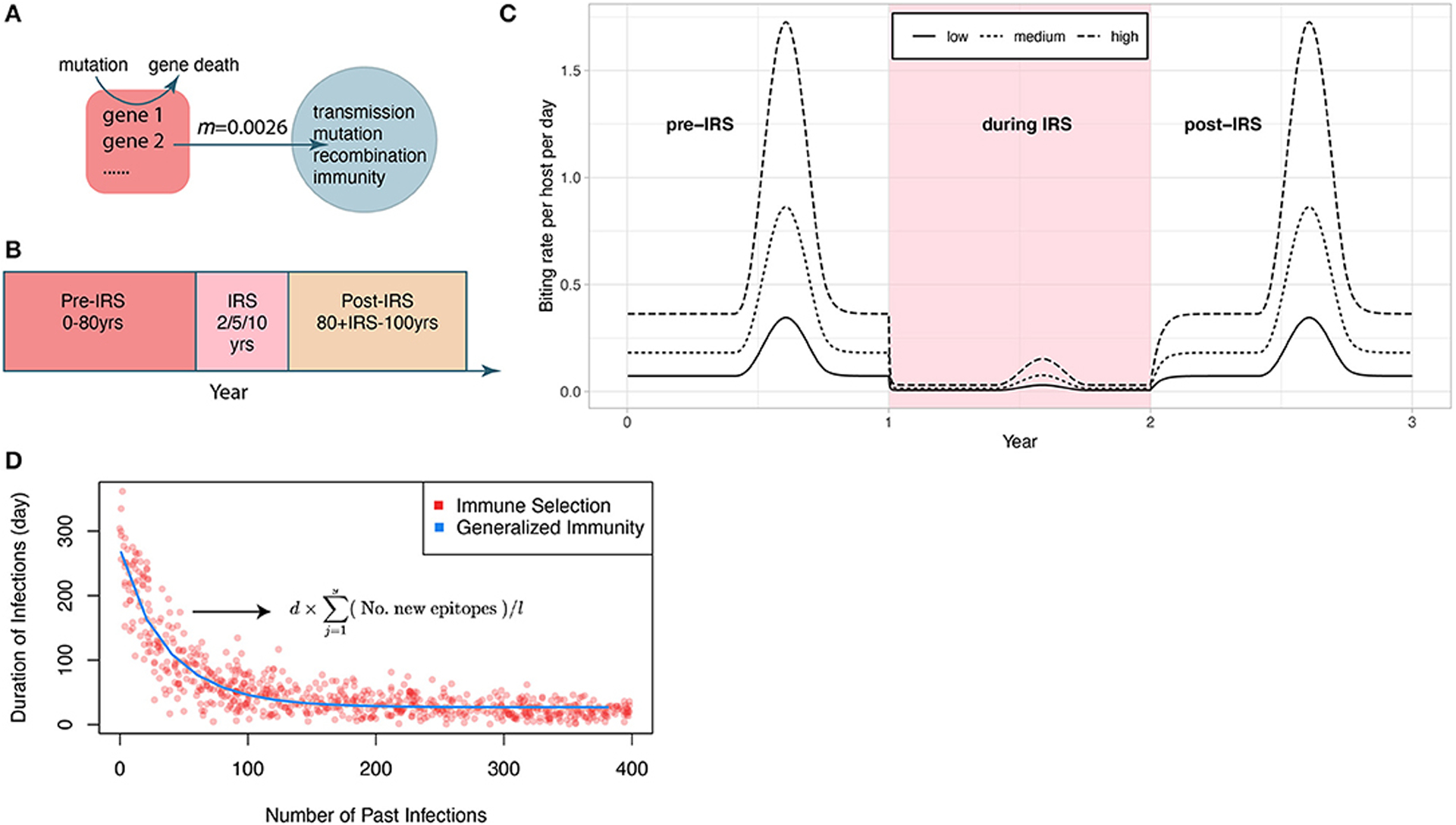
Schematic of the IRS experiment design. **(A)** Initial transmission events and migrant genomes in a local population (blue circle) are sourced from a global pool of genes (red square). Mutation of new genes and the death of existing genes in the global pool occur at the same rate as in the local population (see Methods). The value of the migration rate is inferred from an empirical dataset for a high transmission region in Ghana (see Methods). Individual infections are tracked locally. We also track events of transmission, mutation of genes, recombination within and between genomes, and acquisition and loss of specific immune memory in hosts. **(B)** Each simulation follows three stages (after a burn-in period): a pre-IRS period during which the transmission in the local population reaches a stable state; an IRS period of 2, 5, or 10 years reducing transmission, and a post-IRS period when transmission rates go back to pre-IRS levels. **(C)** Three levels of transmission intensity (biting rates) are explored in the experiments (pre-/post-IRS, low: 44 bites per host per year; medium: 110 bites/h/y; high: 221 bites/h/y). **(D)** The two regimes (with and without NFDS) are ensured to be comparable by specifying the average duration of infection as a function of the number of previous infections in G with a curve fitted to the points generated under S. The expression for the duration of infection under S is given here, and its explanation can be found in the methods (2.2).

We model a local parasite population of size *N*, as well as a global *var* gene pool of size *G*_*p*_ that acts as a proxy for regional parasite diversity (Supplementary Table 1). Parasite genomes can migrate from the regional pool to the local population. Each simulation starts with 20 migrant infections to initiate local transmission (Figure 1A). Because each parasite genome is a repertoire of 60 *var* genes, migrant genomes are assembled from random sampling of 60 *var* genes from the global pool. Each *var* gene is itself a linear combination of two epitopes—the part of a molecule that acts as an antigen and is recognized by the immune system (Rask et al., 2010; He et al., 2018) (*l* in Supplementary Table 1).

We consider seasonal endemic transmission dynamics in which mosquitoes are not represented as agents in the model, but via biting events. Local transmission events are sampled at the total rate of host population size *N*_*h*_ times the biting rate *b*, in which a donor and a recipient host are selected randomly. We implemented seasonality as a fluctuation of mosquito bites, which results from density dependence at the egg and larva stages as a function of rainfall typical of Northern Ghana (availability of breeding sites, White et al., 2011). The specific algorithm to obtain the seasonality of the biting rate was described in detail in Pilosof et al. (2019).

The main modification to the model for this work is how the global pool interacts with local transmission. First, instead of remaining static, the global gene pool in this implementation updates its gene composition at the same mutation rate as that of the local population. Specifically, new genes are generated at a rate equal to the product of local parasite population size and the per-allele mutation rate. Once a new gene is generated, the old gene that it mutates from is removed from the global gene pool. Genomes migrate from the global pool to the local population (Figure 1A). The number of migrant genomes increases in wet seasons and decreases in dry seasons (see details in section Estimation of Migration Rate). Interventions are assumed to be applied at the regional level so that prevalence of the disease is the same across the region, including the local population (Figures 1B,C). Therefore, the proportion of infectious bites from migration is kept the same as that of local transmission.

### 2.2. Parasite Fitness and Duration of Infection

Our main objective was to compare responses of the parasite population to perturbation in the presence and absence of frequency-dependent competition. Specifically, we aimed to compare an “immune selection scenario,” in which immune memory to particular epitopes elicits specific competition between parasite genomes for hosts, to a “neutral scenario” of “generalized immunity” and therefore non-specific competition. In both these scenarios, duration of infection is the relevant trait manifesting the effect of competition for hosts. Parasite fitness is affected by immunity in our model through the duration of infection because shorter duration reduces the probability that a repertoire will be transmitted under a constant transmission rate. In the model with specific immunity, duration of infection depends on the immune history of given *var* epitopes in the host. Hosts gain and lose immunity toward specific epitopes. During infection, expression of the genes composing the given repertoire is sequential and infection ends when the whole repertoire is depleted. The host is considered infectious with the active strain and the expression length *d* of each gene is controlled by host immunity. When a gene is actively expressed, host immunity “checks” infection history to determine whether any of its epitopes have been seen before. Thus, expression length *d* shortens as the number of unseen epitopes out of *l* increases. After the gene is deactivated, the host adds the deactivated gene alleles to its immunity memory. A new gene from the repertoire then becomes immediately active until the end of the repertoire is reached. Therefore, the total duration of infection in a given host with a particular repertoire of *g* genes is given by

$$\text{Total duration}=d\times \sum_{j=1}^{g}(\text{No. new epitopes})/l$$

This is the process that confers an advantage to rare new genes and the parasites that carry them, and a disadvantage to common ones—that is, the frequency-dependent selection. The more similar the epitope composition of two repertoires, the stronger their competition for hosts.

In contrast, the model of generalized immunity retains protection conferred by previous infection but does not consider specific memory toward *var* genes. This is a common implicit assumption of most malaria transmission models, including other ABMs and extensions of the well-known compartmental population models of the SIR type (for Susceptible-Infected-Recovered classes), where infection “consumes” susceptible hosts but as a general and shared resource. In our implementation of generalized immunity, the duration of infection depends on the number of previous infections but not on their specific genetic composition. For a meaningful comparison to the immune selection scenario, we parameterized the function of infection duration with number of previous infections, to match the curve generated by the corresponding immune selection scenario (see He et al., 2018) (Figure 1D).

We refer hereafter to the scenario of immunity-driven frequency-dependent selection as “S” and to that of generalized immunity as “G.” Comparisons between these two scenarios are always made for the same parameter combinations.

### 2.3. Course of a Simulation and Indoor Residual Spraying (IRS) Intervention

Each simulation follows three stages (Figure 1B): (i) a pre-IRS period (0–80 years) during which the local parasite community is assembled and the transmission system reaches a stationary state before the perturbation; (ii) an IRS period of 2, 5, or 10 years during which transmission is decreased; and (iii) a post-IRS period when transmission rates return to pre-IRS levels and the system is allowed to recover (Figure 1C). During the IRS interventions, the effectiveness of insecticides in killing adult female mosquitoes is set to be 100% (Wanjala et al., 2015) and the percentage of sprayed household is set to be 90% (Kigozi et al., 2012; West et al., 2014; WHO, 2015; Coleman et al., 2017). Details on the model can be found in White et al. (2011), and our implementation (in Mathematica) at the GitHub repository https://github.com/pascualgroup/Pf_temporal_networks.

### 2.4. Within-Host Dynamics

The infection and immune history of each host are tracked individually. Upon each biting event, if the donor harbors parasites in the asexual blood stage, then each repertoire has a given probability, *c*, to be transmitted to the mosquito. Maximum transmissibility is set to *c* = 0.5, because not all bites from mosquitoes result in successful transmission from the human host. If the host harbors *n* infections concurrently, each parasite strain experiences a reduced transmissibility equal to *c*/*n*. Hosts can harbor up to 10 concurrent infections. Because parasites must go through the sexual reproduction stage within mosquitoes, during meiotic recombination, *var* repertoires picked up by the mosquito at a bite event may recombine with another genome to produce oocysts that mature into sporozoites. Since each oocyst is generated from recombination between two parental genomes, if a mosquito picks up *n* genomes, each genome has a probability 1/*n* of recombining with itself, producing the same offspring genome, and a probability 1 − 1/*n* of recombining with a different genome, producing recombinants. To generate a recombinant, two sets of genes are pooled from the original genomes, and a random set is sampled. Since *var* genes are distributed across most of the chromosomes, there is a substantial shuffling of genes during meiotic recombination. Although it is possible that some genes are linked and do not recombine, we do not know the exact physical locations of these genes. It is therefore a simplification in our model to take a random sample from two pooled genomes. This sexual recombination process creates variation at the genome level. The total number of *var* repertoires passed onto the receiving host is kept the same as that obtained from biting the infectious donor host.

Once in the blood, each infection progresses with the sequential expression of the *var* genes in random order. During an infection, novelty at the gene level can be generated via mutation or recombination between *var* genes within the same genome. Part of the life cycle of *Plasmodium falciparum* occurs in the liver; we therefore implement a 14 day delay to mimic the transition from the liver stage to the blood stage, where the parasite becomes infectious and expression of the *var* genes is initiated.

Hosts gain and lose immunity toward specific epitopes (*l*_*i*_), which we implement with a counter to track the boosting of immunity (the number of times a host has been exposed to a given variant) and the loss of immunity. After expression of a given *var* gene, the host gains full protection toward the epitopes in the gene that was previously expressed. However, the immunity loss rate for a specific epitope depends on the number of times the host has been exposed to it. For example, if the host was infected by a parasite that contained two *var* genes, each of which contained an allele encoding epitope *x*, the counter for this epitope will increase by two. The counter decreases by one at the immunity loss rate of 1/1,000 per day (Collins et al., 1968). When the counter value becomes 0, the host loses protection against the given epitope.

### 2.5. Estimation of Migration Rate

We use empirical data of *var* genes, hyper-diverse markers that provide a higher resolution for recent migration events, to infer the rate of gene exchange between populations and to obtain a reasonable estimate for migration rates that allows us to implement an open transmission system. We used Jost’s *D* measure of population divergence (Equation 12 in Jost, 2008) to consider highly diverse genetic markers, and to compare *var* gene composition within and between two field sites for which molecular sequences were previously obtained: Soe and Vea/Gowrie in the Bongo District of Ghana at the end of the wet (high transmission) season (Tiedje et al., 2017; He et al., 2018). Using Equation (22) in Jost (2008), we estimated *m*, the migrant proportion (percentage of migrant bites relative to local transmission events), by dividing *D* by the mutation/recombination rates of the *var* genes per generation (5.3e-6, Claessens et al., 2014). Thus, the migration rate per day is the product of *m* * *b*.

### 2.6. Event Scheduling in the Stochastic Model

The simulation is an ABM implemented with a modified Gillespie algorithm called next-reaction method (Gillespie, 1976), which is a computationally-efficient way to model stochastic dynamics. The algorithm takes a set of events (e.g., infection, biting) and assigns for each event the time of its next occurrence by sampling an exponential distribution with a mean of 1 over its rate, to implement a Poisson process. The next event is then chosen to be the one with the closest event time.

In our simulation, global events include local transmission from biting events, new transmission from migrant *var* repertoires, and birth and death of hosts. In the numerical implementation of the simulation, all possible future events are stored in a single event queue along with their putative times. When an event occurs, it may trigger the addition or removal of future events on the queue, leading to a re-ordering of events, or changes of their rates, and to a recalculation of their putative time. The next-reaction method optimizes the Gillespie first-reaction method (Gillespie, 1976) and allows for faster simulation times, as it targets changes of rate events to a given subset (specified by the structure of the queue).

### 2.7. Experimental Design

We explored how initial gene pool size, transmission rate and the duration of an IRS intervention influence the system’s persistence to, and recovery from, intervention under G and S. Specifically, we set: (i) initial gene pool sizes (*G*_*p*_) of 1,200, 1,800, 2,400, 3,600, 4,800, 7,200, 9,600, 14,400, and 19,200; (ii) three levels of transmission intensity (biting rates; *b*): 44, 100, and 221 bites per host per year, corresponding to “low,” “medium,” and “high” transmission (Figure 1C); and (iii) IRS lasting for 2, 5, or 10 years. This design has 81 sets of parameter combinations and we ran 50 replications for each combination. We calculated the probability of extinction, effectively measuring persistence, as the proportion of the 50 replications in which the parasite populations crashed before lifting the IRS.

### 2.8. Sampling and Gene Similarity Networks

During each simulation, summary statistics including prevalence, multiplicity of infection (MOI; number of genomes within a host), and genetic and allelic diversity, were calculated every 30 days. In addition, 100 infected hosts were randomly sampled to analyze parasite diversity patterns. To evaluate similarity of parasites in the population, pairwise type sharing (PTS) was calculated between all repertoire pairs (regardless of the host in which they are encountered) as *PTS*_*ij*_ = 2*n*_*ij*_/(*n*_*i*_ + *n*_*j*_), where *n*_*i*_ and *n*_*j*_ are the number of unique alleles (corresponding to epitopes) within each repertoire *i* and *j* and *n*_*ij*_ is the total number of alleles shared between repertoires *i* and *j* (Barry et al., 2007). In addition, similarity networks based on the *var* composition were built to investigate changes of parasite genetic population structure through interventions. We calculated genetic similarity of repertoire *i* to repertoire *j* as *S*_*ij*_ = (*N*_*i*_ ∩ *N*_*j*_)/*N*_*i*_, where *N*_*i*_ and *N*_*j*_ are the number of alleles unique for repertoires *i* and *j*, respectively (the genetic similarity of repertoire *j* to repertoire *i* was calculated as *S*_*ji*_ = (*N*_*i*_ ∩ *N*_*j*_)/*N*_*j*_). Unlike PTS, network edges encode a directional measure that represents the asymmetric competition between repertoires, resulting from different numbers of unique alleles (He et al., 2018).

### 2.9. Calculation of Network Properties and Principal Component Analysis

We calculated 36 network properties to compare the changes in network structure between the immune selection and neutral scenarios. These properties include metrics of transitivity, degree distributions, component sizes, diameters, reciprocity, and proportion of 3-node graph motifs (see Supplementary Table 1 in He et al., 2018 for a complete list of properties and definitions). In the network analyses we retained only edges with values within the 95% percentile to focus on the strongest interactions of current competition between strains (He et al., 2018; Pilosof et al., 2019). To minimize the influence of sample size differences across time due to changes in mean MOI, network properties were calculated by resampling 100 repertoires randomly from the original network. Principal component analysis (PCA) was performed on normalized and centered network properties across time and selection regimes per parameter combination, to inspect overall trends in network structure.

## 3. RESULTS

### 3.1. Populations Under Immune Selection Are More Persistent

**FIGURE 2 |:**
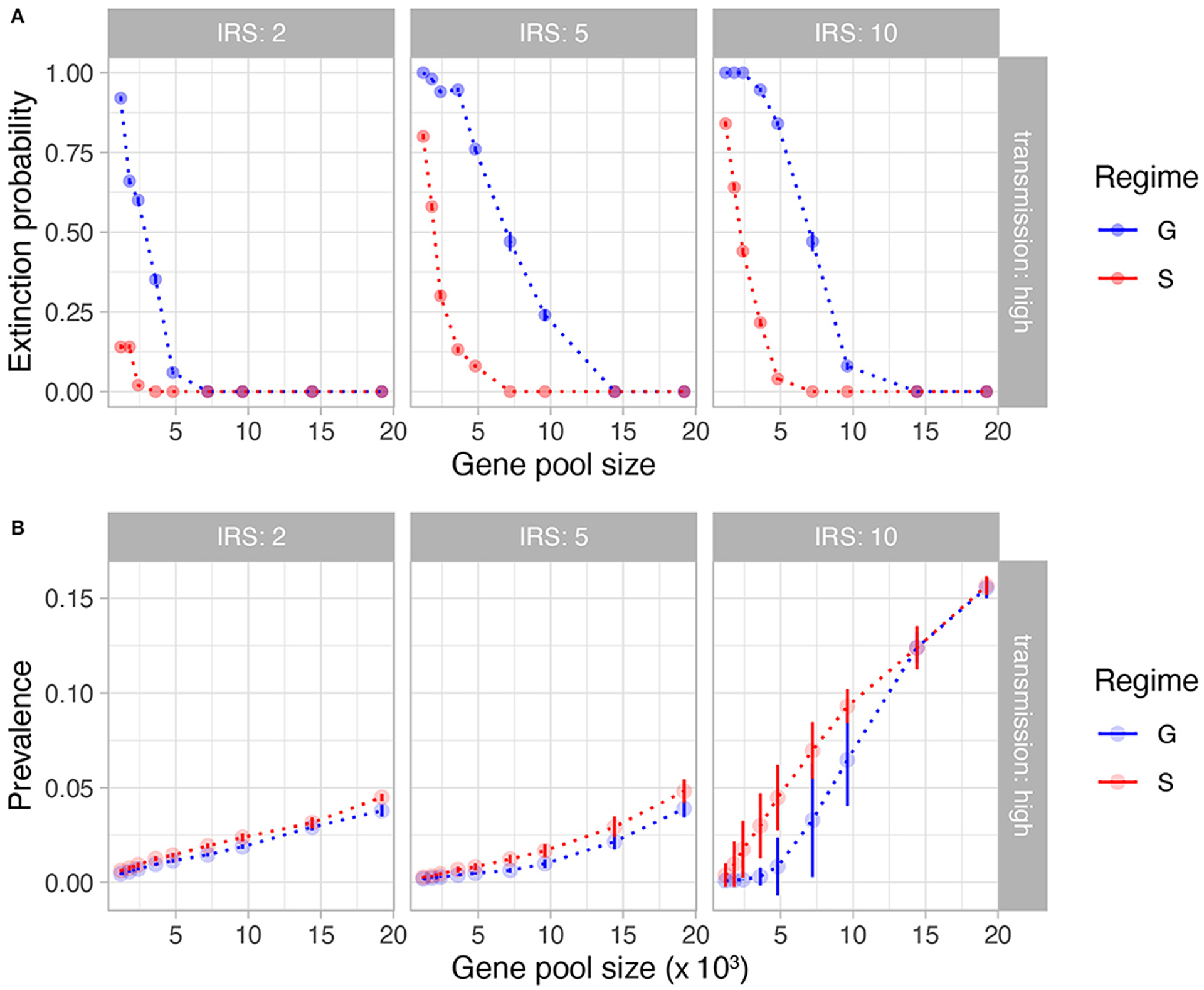
Extinction probability **(A)** and mean prevalence during IRS **(B)** as a function of initial gene pool size and lengths of IRS (columns). Shown here are the results from high transmission intensities. See Supplementary Figure 1 for results from low and medium transmission intensities. In **(A)**, each point is the proportion of simulation runs for the given parameter combination (out of 50 runs) that crashed before the IRS was lifted. In **(B)**, each point represents the mean value (bars represent standard deviation) of prevalence for those simulation runs that survived the IRS for the given parameter combination. Blue and red colors depict generalized immunity (G, the neutral model) and specific immunity (S, NFDS), respectively. It is clear that despite comparable prevalence, the parasite population is more persistent in the immune selection scenario.

The interventions implemented in the model reduce the transmission rates by about 90% during IRS. Not surprisingly and especially for the long-lasting interventions, the stochastic simulations are prone to extinction. We found a lower extinction probability under specific immunity (S) than under generalized immunity (G) for the same parameters (Figure 2A, Supplementary Figure 1A). As expected, longer interventions lead to higher extinction rates, especially under low transmission rates. A higher initial pool size of genetic diversity ensures a lower extinction probability. Pool sizes larger than 10,000 *var* genes, which represent high transmission endemic regimes in sub-Saharan Africa, experience significantly less extinction, even after 10 years of sustained intervention. Interestingly, the difference in extinction probability between S and G does not reflect a trivial effect of overall population size of the parasite: the mean prevalence is comparable during IRS for S and G, only slightly higher for the former, indicating that other factors are responsible for the higher persistence under NFDS (Figure 2B, Supplementary Figure 1B).

### 3.2. Persistence Is Enhanced by Repertoire Dissimilarity Under NFDS

**FIGURE 3 |:**
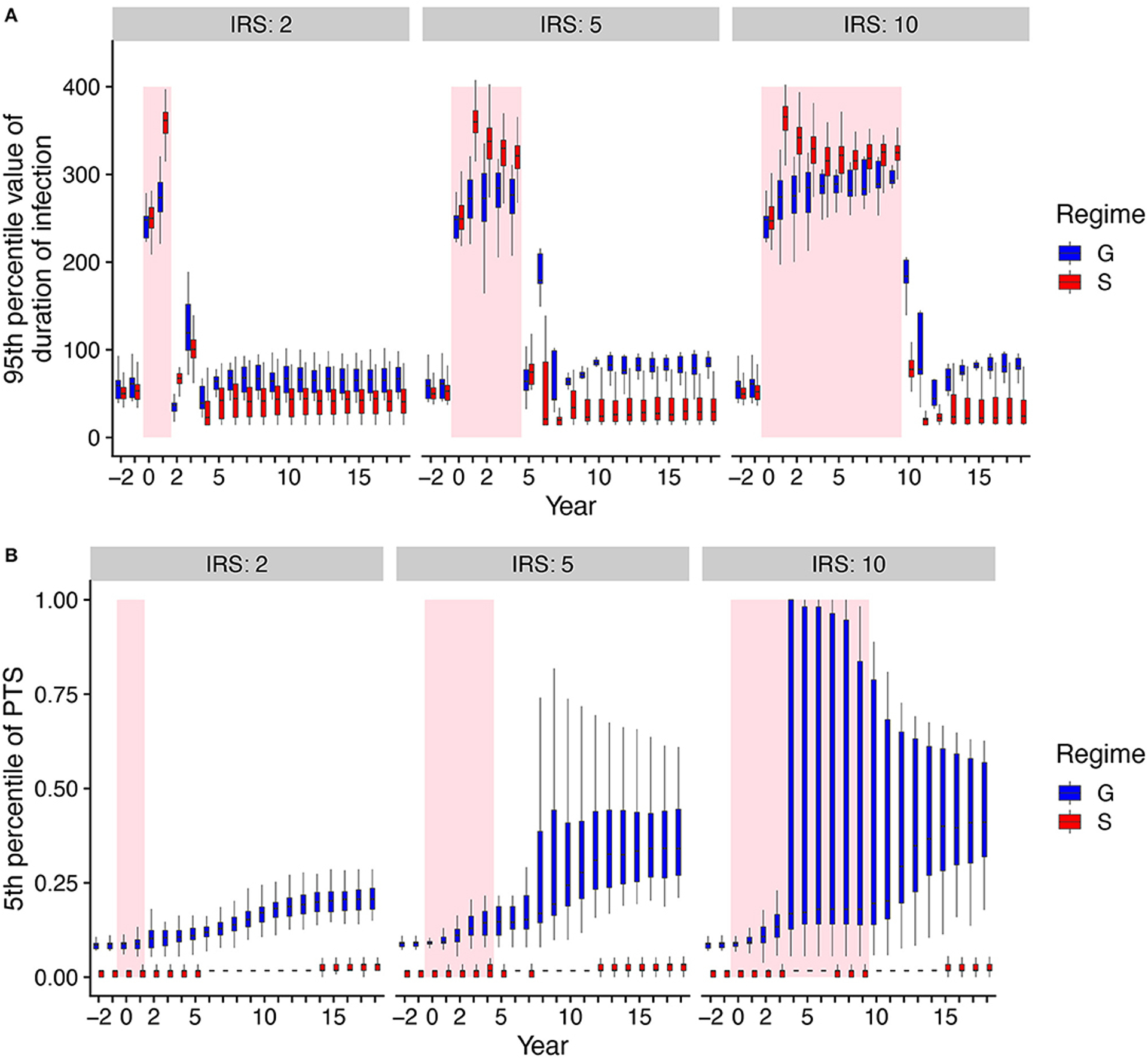
Comparisons of duration of infection and mean PTS over time under high transmission between the two regimes with and without NFDS. Boxplots summarize the distribution among replication runs of the 95th percentile of duration of infection **(A)**, and the distribution of the 5th percentile of PTS between var repertoires **(B)**. We therefore focus on the longest periods of infection duration and correspondingly, the most dissimilar parasites. Shaded areas indicate the intervention (IRS) period. Negative years correspond to times prior to IRS.

To investigate the origin of the difference in persistence as measured by the probability of extinction, we consider the epidemiological parameter that confers the fitness difference to the parasite, namely the duration of infection in the human host. As is typical in epidemiology, the reproductive number of the parasite is proportional to the product of the transmission rate and the duration of infection. In our model, immunity modifies only duration of infection as genes encoding for antigenic variants which have been seen before are not expressed. We note that we set the mean duration of infection with the number of previous exposures under G to exactly match that under S before IRS for a specific parameter combination (Figure 1D). Therefore, the mean values of duration of infection under G and S are similar before the IRS by construction, with the values increasing significantly during IRS (Supplementary Figure 2). When the longest infections are considered by comparing the top 5% of the distribution of infection duration, we observe higher values under S during IRS obtained in less than a year (Figure 3A, Supplementary Figure 3). Longer lifetimes of infection at the tail of the distribution provide a buffer against extinction by conferring a higher probability of survival to parasites during the low transmission period imposed by the intervention.

**FIGURE 4 |:**
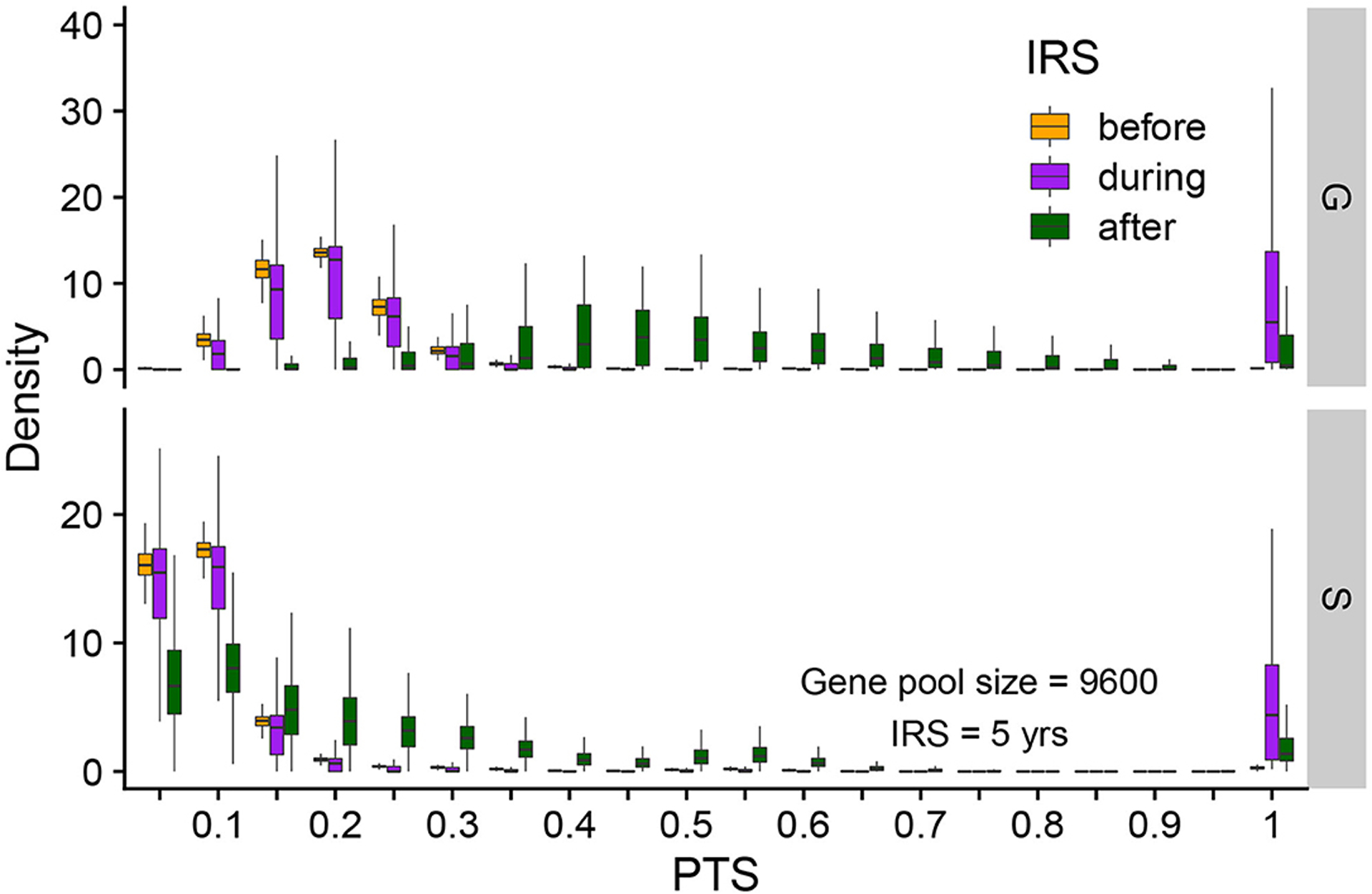
Distribution of pairwise PTS for *var* repertoires before (orange), during (purple), and after (green) intervention under S and G. PTS values are binned into 40 equally-sized bins (of 0.05). Each box shows the variation of PTS values across different runs. Summarized here are runs from a gene pool size of 9,600, high transmission and a 5-year intervention. The PTS distributions differ between G and S. After intervention, the PTS distribution in G drifts away from its initial state, whereas that in S tends to go back to its pre-IRS state.

To further understand the extinction patterns, we therefore need to explain the longer durations of infection at the tail of their distribution under NFDS. We turn to the structure of similarity between parasites as reflected by the distribution of pairwise type sharing (PTS) between *var* repertoires (Figures 3B, 4, Supplementary Figure 4). Figure 4 shows the PTS distributions before, during, and after the IRS intervention for the two scenarios. The shapes between the two scenarios differ substantially at low PTS, reflecting clear patterns of high dissimilarity in S. In particular, NFDS generates a monotonically decreasing distribution with the highest frequencies found at the lowest overlap. In contrast, under G the distribution exhibits a mode, with the highest frequency for some intermediate overlap. Due to decreased diversity during IRS, the proportion of repertoire pairs sharing more epitopes increases for both scenarios, and the whole distribution moves toward higher overlap (i.e., higher PTS) (Figure 4). However, the change is less pronounced under S, especially for the maintenance of dissimilar repertoires (Figure 3B). When the intervention is lifted, the distribution of G shifts significantly toward increased similarity, while the bimodal shape of the PTS distribution in S is maintained, including a considerable fraction of highly dissimilar strains. Since diversity under G does not influence transmission by construction, the change in PTS reflects solely parasite population fluctuations and bottleneck effects during and after IRS. In contrast, the maintenance of low PTS values that are comparable to pre-IRS levels under S is indicative of variant-specific immune selection at work: even under reduced diversity, repertoires are maintained as different from each other as possible, resulting in some longer infections than for a randomly assigned gene composition. A larger fraction of the parasite population with highly dissimilar repertoires generates higher heterogeneity in duration of infection, including longer duration, increasing the persistence during intervention under S.

### 3.3. Prevalence Recovers to a Lower Level Than Pre-intervention Under NFDS Because of Diversity Loss

**FIGURE 5 |:**
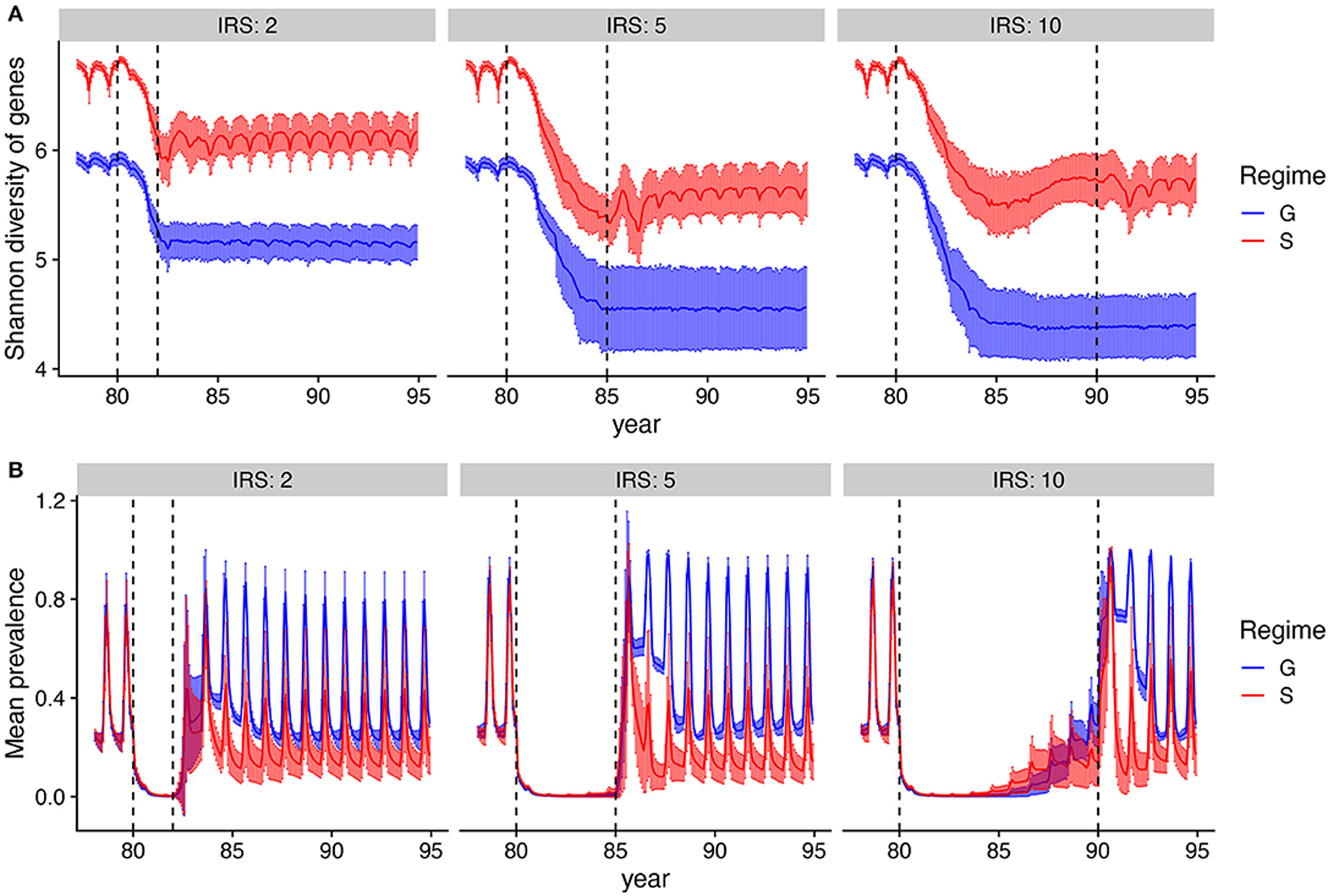
Dynamics of genetic diversity and prevalence during and after IRS. **(A)** Mean Shannon diversity (solid line) and standard deviation (error bars) of var gene types are tracked over time. Shaded areas show the extent of the variation across runs. **(B)** Mean changes in prevalence (solid line) and its variation (error bars) over time. After IRS, prevalence returns back to pre-IRS stage under generalized immunity (G), whereas it gravitates to a lower level under specific immunity (S). Parameters: gene pool size equals 9,600 and transmission rate is high. Only runs that persisted after IRS are included.

We focus next on the recovery from intervention in terms of both antigenic diversity and prevalence. As expected, both antigenic diversity and prevalence decrease with intervention, and longer IRS leads to a higher reduction in antigenic diversity than the shorter 2-year IRS. Post-intervention, diversity settles on a new equilibrium, that is lower than the pre-intervention one (Figure 5). Although new genes should be strongly preferred under S (but not G), their generation from mutation, ectopic recombination, and immigration, is likely too slow to rebuild locally to pre-intervention levels given the parameters we use. Moreover, the intervention was implemented to also affect the *var* gene diversity of the regional pool, hindering regeneration of diversity via migration. We touch upon this point further in the Discussion.

All simulations that persist the intervention show a comparable initial rebound of prevalence in the months that immediately follow the control period. The rebound for G is to the same prevalence than that before intervention, as the transmission rate has been restored and the duration of infection only depends on the exposure rate. In contrast, under S, after an initial overshoot—which is a result of a large human population that is not immune to the parasite due to limited infections during IRS—mean prevalence attains lower values (Supplementary Figure 5, Figure 5B), because the decreased *var* gene diversity induces a shorter duration of infection. The rebound of diversity under S is therefore a result of immune selection and not a larger parasite population size post-IRS. The recovery of prevalence post-intervention can only be achieved to a lower level than pre-intervention, given that the *var* gene diversity itself cannot be completely rebuilt.

### 3.4. The Structure of Genetic Diversity Is Restored After Intervention Under NFDS

Until now we have mainly addressed the connection between antigenic diversity and perturbation in relation to similarity (PTS), as one feature of the structure of diversity. We consider next the structure of similarity between *var* repertoires more broadly by applying an analysis of network features (sensu He et al., 2018). In these networks, the nodes are repertoires and the directional links quantify the degree of overlap in *var* gene identity between them. The degree of overlap between two repertoires is a measure of the strength of competition between them for human hosts (i.e., intensity of competition positively correlates with similarity). Therefore, we only retain the links above a given cutoff because it is for these most similar repertoires that we expect the selection against recombinants to be the strongest, and the evidence of frequency-dependent selection to be most apparent (see He et al., 2018). We know from our previous work that we can use ensembles of network features to differentiate between populations of parasites assembled under S and G. Here, we compare the overall similarity structure between these regimes before, during and after intervention, to ask whether and how quickly the effect of NFDS is restored after intervention, as we expect it to be relaxed under the decreased transmission of intervention.

**FIGURE 6 |:**
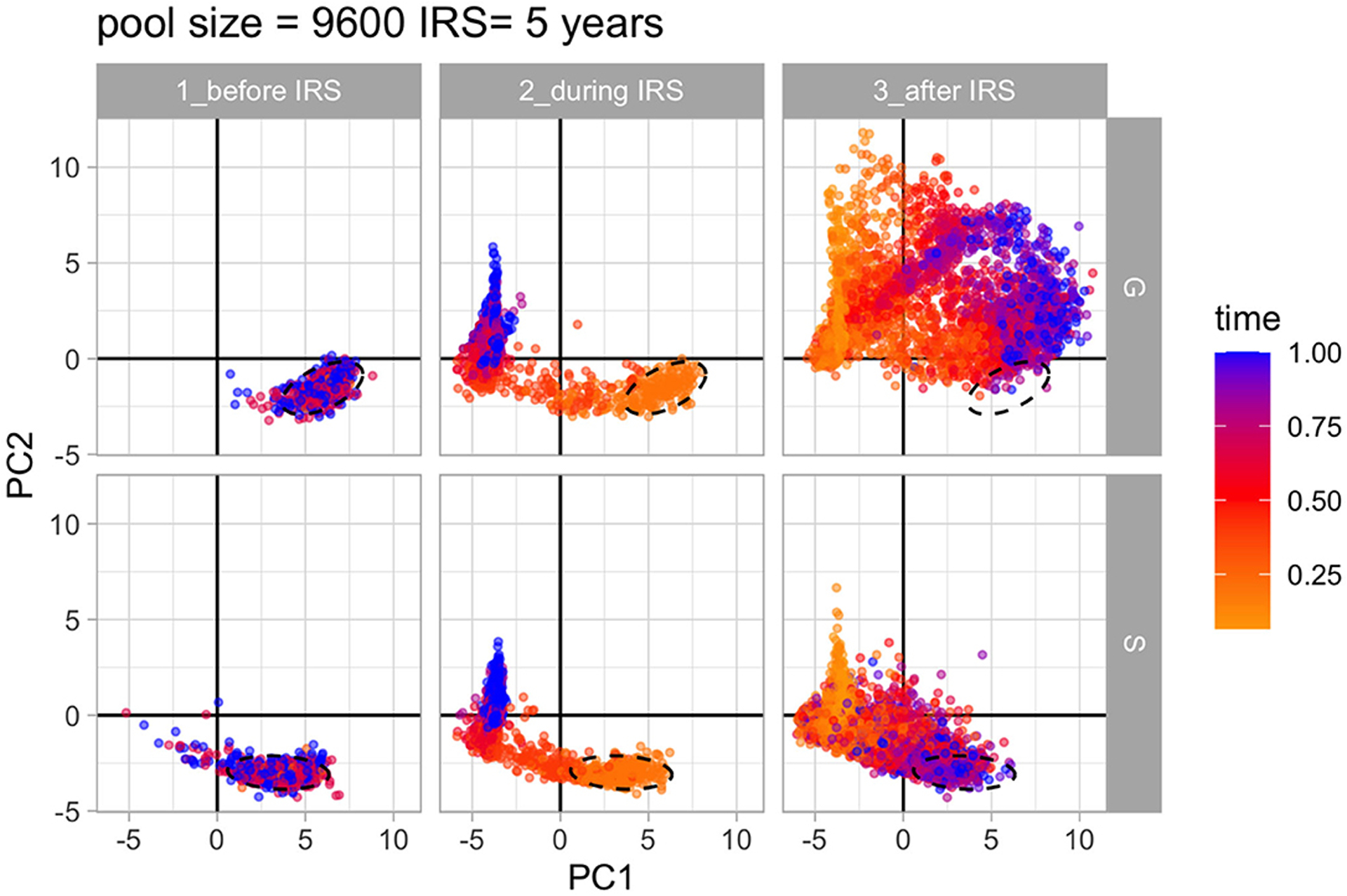
Principal component analysis of 37 network properties of *var* repertoire similarity networks. PCA over time shows how the structure of diversity changes in the 2D space of the PCs for S and G under high transmission. The colors of the networks represent their relative time within the specific time period (i.e., before, during and after IRS) in the simulation. Ellipses show the location of network properties for pre-IRS layers. Parameters: gene pool size equals 9,600 and IRS lasts for 5 years.

The similarity structure for an ensemble of simulations can be visualized in the 2-D space defined by the two major axes of variation of PCA (Methods). Figure 6 and Supplementary Figure 6 show that the networks assembled under S and G do indeed differ and occupy different regions in that space before intervention. Their structure changes and becomes similar for the intervention period as selection/competition is relaxed. Nevertheless, under S the structure is quickly restored after intervention, whereas under G this is not the case, with networks drifting further away from each other and from their initial structure.

The network features contributing the most to the classification of the similarity structure are shown in Supplementary Figure 7. These include in particular the 3-node motifs and reciprocity that characterize local structures and divergence between communities within networks that characterize global structures. The properties distinguish the more tree-like local structure of neutrality from that of limiting similarity and more symmetric or balanced diversity of selection (He et al., 2018). The action of frequency-dependent selection on similarity structure is what eventually maintains and restores the distributions of overlap described earlier (Figure 4), which explains the longer duration of infection and therefore the higher probability of persistence during intervention.

## 4. DISCUSSION

Understanding the relationship between structure and persistence in diverse communities is a long-lasting and on-going effort. This relationship likely depends on the processes that generate diversity and assemble its structure. High-transmission endemic malaria constitutes a relevant host-pathogen system to investigate this relationship in the context of negative frequency-dependent interactions, which stabilize coexistence and structure diversity via patterns of limiting similarity in antigenic space. Here, we show that NFDS is important not only for coexistence and structuring *P. falciparum* populations, but for the response of these populations to perturbations.

In the malaria system, limiting similarity emerges from the dominant force behind the large antigenic diversity—namely NFDS from competition of parasites for hosts, mediated by adaptive specific immunity. We specifically asked about the connection between the population structure of pathogen genetic diversity and stability to press perturbations that reduce the abundance of the parasite. We have shown that repertoire populations assembled under selection exerted by host immunity are more persistent to intervention than those assembled under a neutral model with a generalized form of immunity. We have linked this persistence to the larger fraction of highly dissimilar repertoires that provides the parasite with a way to infect non-immune hosts. Interestingly, the network analyses of parasite similarity reveal that the effect of NFDS is quickly restored after the intervention is lifted, which indicates that the process acting to maintain patterns of limiting similarity acts strongly on the parasite population. This quick rebound provides a different angle on the stability of the system.

Although the strength of NFDS is quickly restored to maintain low similarity between strains, the *var* gene diversity in our simulations slowly rebounds but does not recover to pre-intervention levels. In part, this is the consequence of a regional intervention where the processes generating new genes are not fast enough to rebuild the original local and regional diversity. The time scale at which new genes can be generated and the selective advantage they represent will be critical parameters determining the speed of diversity recovery, but also whether it can practically rebuild to the original pre-intervention levels. This aspect of the system’s recovery will be investigated in future work, in light of the recently introduced concept of a threshold for the accumulation of antigenic diversity, we named the innovation number *R*_*div*_, whose critical value is one (He and Pascual, 2021). Here, we have considered parameter ranges representative of those in nature, although the values of ectopic recombination rates have been measured only *in vitro* (Claessens et al., 2014).

Our theoretical results suggest that competition for hosts can hamper malaria interventions, by operating to maintain the most dissimilar repertoires (Chen et al., 2011; Ruybal-Pesántez et al., 2017). This is in line with the challenge of malaria elimination that characterizes high transmission regions with *var* gene diversity levels comparable to those considered here, even after intense eradication campaigns. For example, Coleman et al. (2017) showed that after lifting a 7-year long IRS effort, transmission intensity (i.e., entomological inoculation rate, EIR) increased from 30 to 90 infectious bites per person per month in only 2 years, despite a consistent reduction in both EIR and sporozoite rates compared to a nearby control site during the intervention. Thus, transmission persists and prevalence rebounds.

Our model considers a local population embedded within a regional pool that provides a source of genetic variation. This approach is a first step toward developing a more comprehensive theory because in nature malaria is transmitted within and between local human populations, effectively creating a metapopulation of parasites. Therefore, processes that operate on metapopulations, such as dispersal and source-sink dynamics, may influence both the assembly of parasite populations and their stability in addition to local selection. For our purpose, this metapopulation context is particularly relevant in creating a vast pool of genetic variation as documented for endemic regions over larger spatial scales (Day et al., 2017; Tonkin-Hill, 2020) than those of local transmission, and longer temporal scales than those of the intervention we implement here. Addressing metapopulation dynamics explicitly for this highly diverse system would be however computationally extensive. Our approach relies on the initial compromise of a global pool typical of many assembly models in ecology.

While NFDS is a form of balancing selection, we note the difference in the organizational level when referring to genes (such as MHC) or genotypes (combinations of genes). While NFDS can operate at both levels (Takahata, 1990), in this study we have focused on the genotype level. We conjecture that coexistence under NFDS should apply to other ecological systems where frequency-dependent interactions and resulting selection play an important role in the coexistence of a large number of entities, and possibly concurrently in the establishment of a large pool of underlying genetic/trait diversity (Pascual, 2020). For example, negative density-dependent mortality of offspring in rainforests arises from interactions with natural enemies and mutualists (Janzen, 1970; Mangan et al., 2010; LaManna et al., 2017; Schroeder et al., 2020), and traits associated with such mortality concern phytochemistry (Forrister et al., 2019). In microbial systems, where diversity is typically large, NFDS generates populations composed of multiple coexisting strains and mediates population structure as well as response to vaccination (Corander et al., 2017). Moreover, similarly to malaria, interactions of microbes with their viral predators mediated via the CRISPR adaptive immune system, result in a modular network structure. This structure occurs because viruses with segments of DNA/RNA recognized by the bacterial CRISPR system that are rare, have an advantage over those with segments that are frequent (Pilosof et al., 2020). We speculate that in these and other cases, the resulting community structure should increase stability by reducing overlap between species/genotypes in trait space, allowing for an overall better ability to respond to perturbations. This conjecture is supported by empirical evidence in other pathogen systems for the importance of frequency-dependent interactions in structuring diversity (Corander et al., 2017; Harrow et al., 2021), and by recent results showing predictive effects on strain structure following vaccination (Azarian et al., 2019; McNally et al., 2019). One way to advance relevant theory aiming to explore this hypothesis is via the combination of ABMs and network analyses of the resulting diversity structure. Such theory can guide similar network analyses of complex data sets to address the role of non-neutral processes and associated patterns of similarity based on relevant traits.

## Supplementary Material

Supplementary_Material

## Data Availability

The agent-based stochastic simulator of malaria dynamics and the processing scripts to reproduce all the figures are stored and annotated at the GitHub repository: https://github.com/pascualgroup/frontiers_var_processing_code.
