# Supplementary material for: Frequency-Dependent Competition Between Strains Imparts Persistence to Perturbations in a Model of Plasmodium falciparum Malaria Transmission
Source: Front Ecol Evol. Author manuscript; Available in PMC 2022 Apr 15. (PMC9012452; doi:10.3389/fevo.2021.633263)
Supplement: Supplementary_Material [file NIHMS1794619-supplement-Supplementary_Material.pdf]

Frequency-dependent competition between strains imparts persistence to perturbations in a model of *Plasmodium falciparum* malaria transmission.

Qixin He, Shai Pilosof, Kathryn E. Tiedje, Karen P. Day, Mercedes Pascual

1 SUPPLEMENTARY TABLES AND FIGURES

Table S1. Epidemiological and genetic parameters used in stochastic simulations. Note that the size of the global *var* gene pool changes as the simulation progresses (see Methods)

Symbol	Type	Description	Values	Unit
G_p	number	*var* gene pool size at the beginning of the simulation	[1200, 1800, 2400, 3600, 4800, 7200, 9600, 14400, and 19200]	
l	number	epitopes	G_p/10	per locus
d	time	length of gene expression	6	days
b	rate	contact rate	low = 44<br>medium = 100<br>high = 221	bites per person per year
δ	rate	immune memory loss rate	0.001	per locus per day
m	probability	migrant proportion	0.26	percentage of local transmission
μ	rate	mutation rate	1.42e-08	per parasite per locus per day
r	rate	recombination rate	1.8e-07	per parasite per gene per day
N_h	number	host population size	10,000	
c	proportion	maximum transmissibility	0.5	per bite

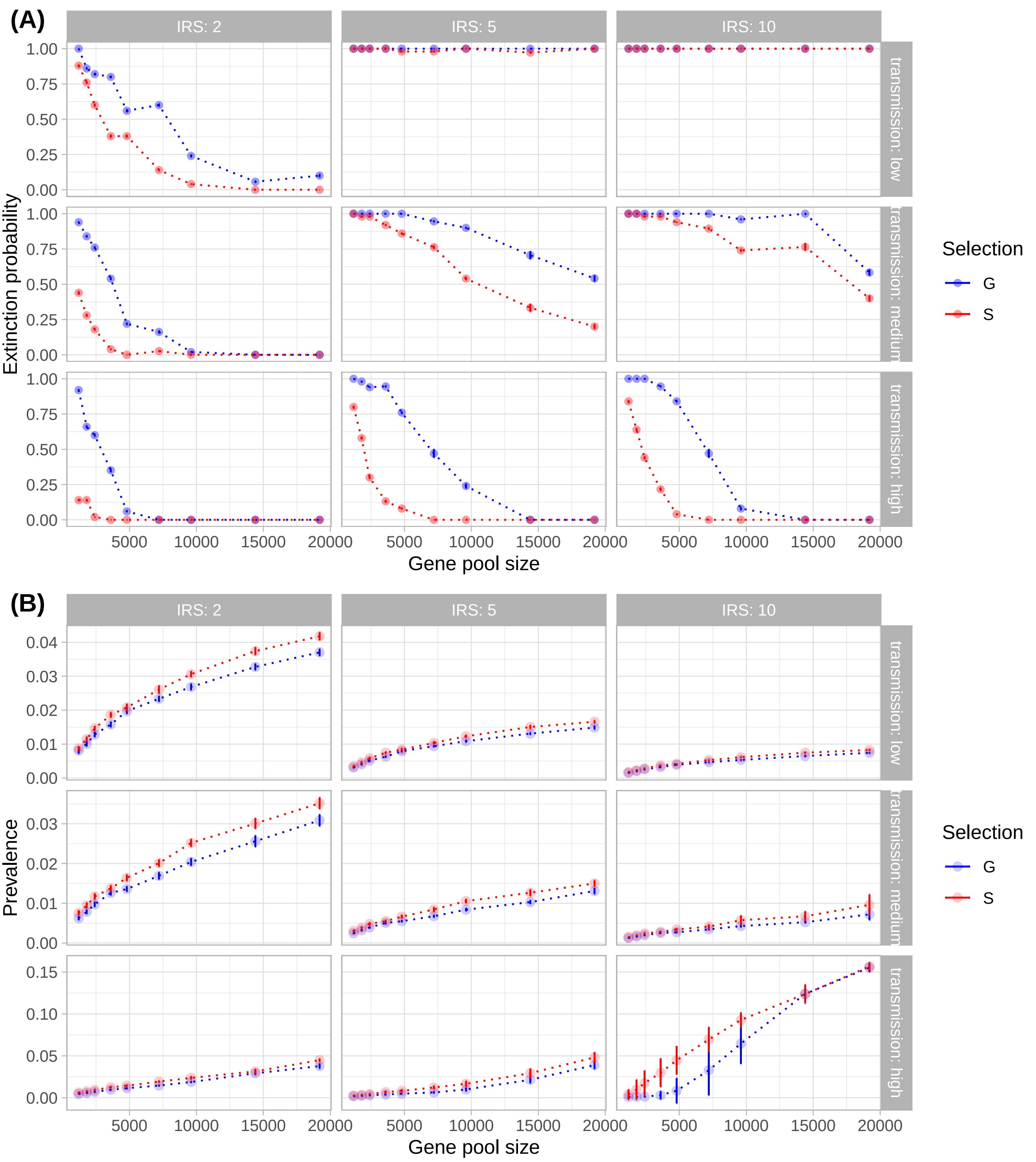

Figure S1. Extinction probability (A) and mean prevalence during IRS (B) as a function of initial gene pool size, under different transmission intensities (rows), and lengths of IRS (columns). In (A), each point is the proportion of simulation runs for the given parameter combination (out of 50 runs) that crashed before the IRS was lifted. In (B), each point shows the mean value (bars represent standard deviation) of prevalence for those simulation runs that survived the IRS for the given parameter combination. Blue and red colors depict generalized immunity (G, the neutral model) and specific immunity (S, NFDS), respectively. It is clear that despite comparable prevalence, the parasite population is more persistent in the immune selection scenario.

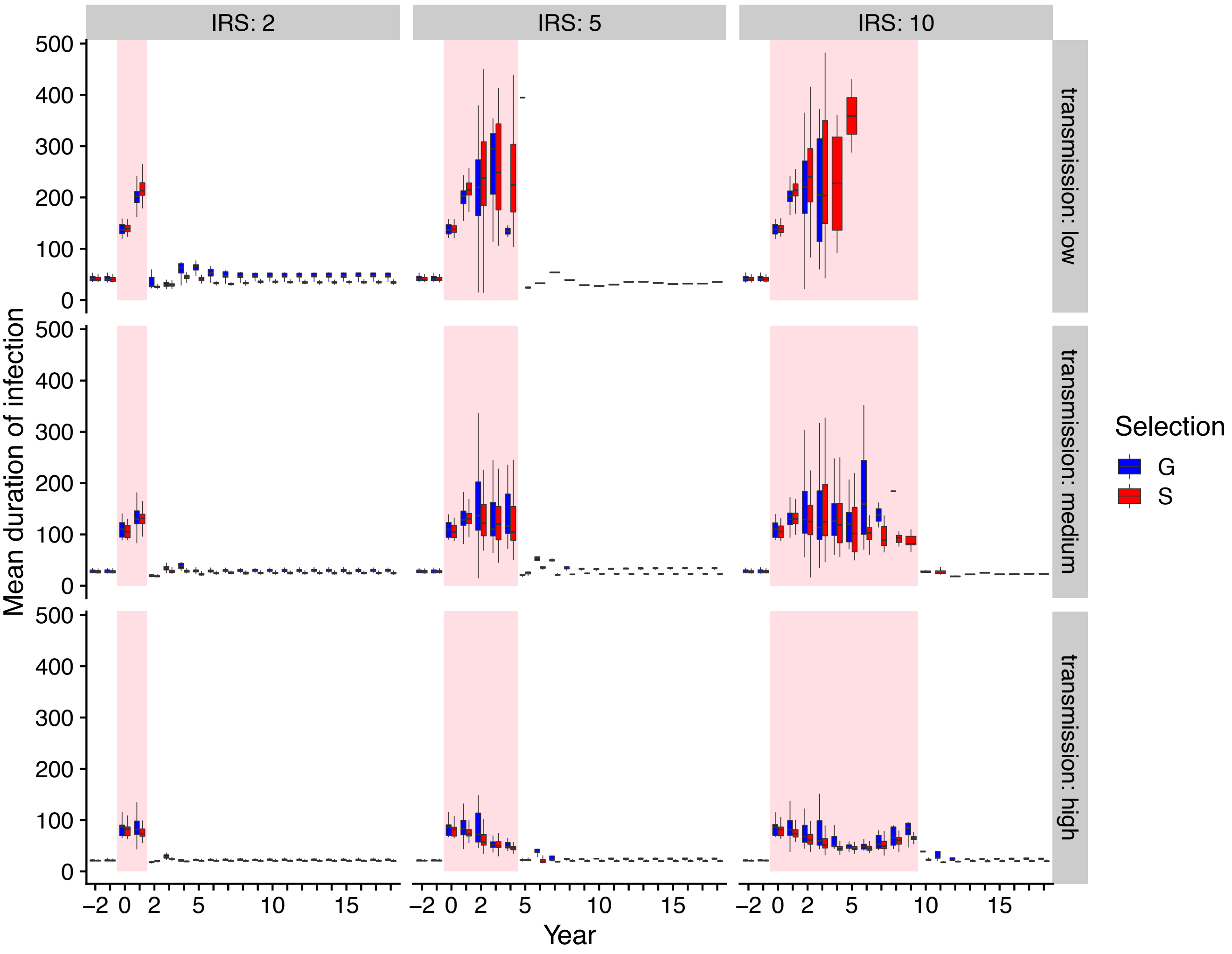

Figure S2. Comparisons of the mean duration of infection over time between different selection regimes given different transmission intensities. Box-plots summarize the distribution of the mean duration of infection across different gene pool sizes and among replication runs. Highlighted areas indicate the intervention (IRS) period. Shaded areas indicate the intervention (IRS) period. Negative years correspond to times prior to IRS.

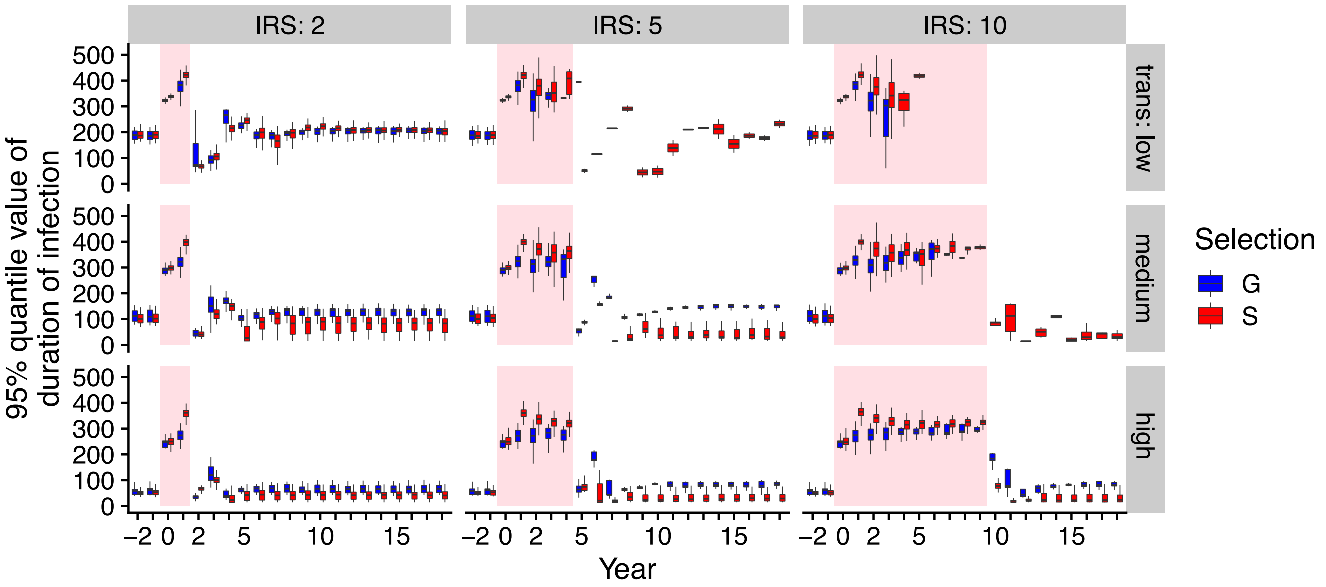

Figure S3. Comparisons of the 95th percentile of duration of infection over time between S and G given different transmission intensities. Box-plots summarize the distribution of duration of infection in its 95th percentile across different gene pool sizes and among replication runs. Highlighted areas indicate the intervention (IRS) period. Shaded areas indicate the intervention (IRS) period. Negative years correspond to times prior to IRS.

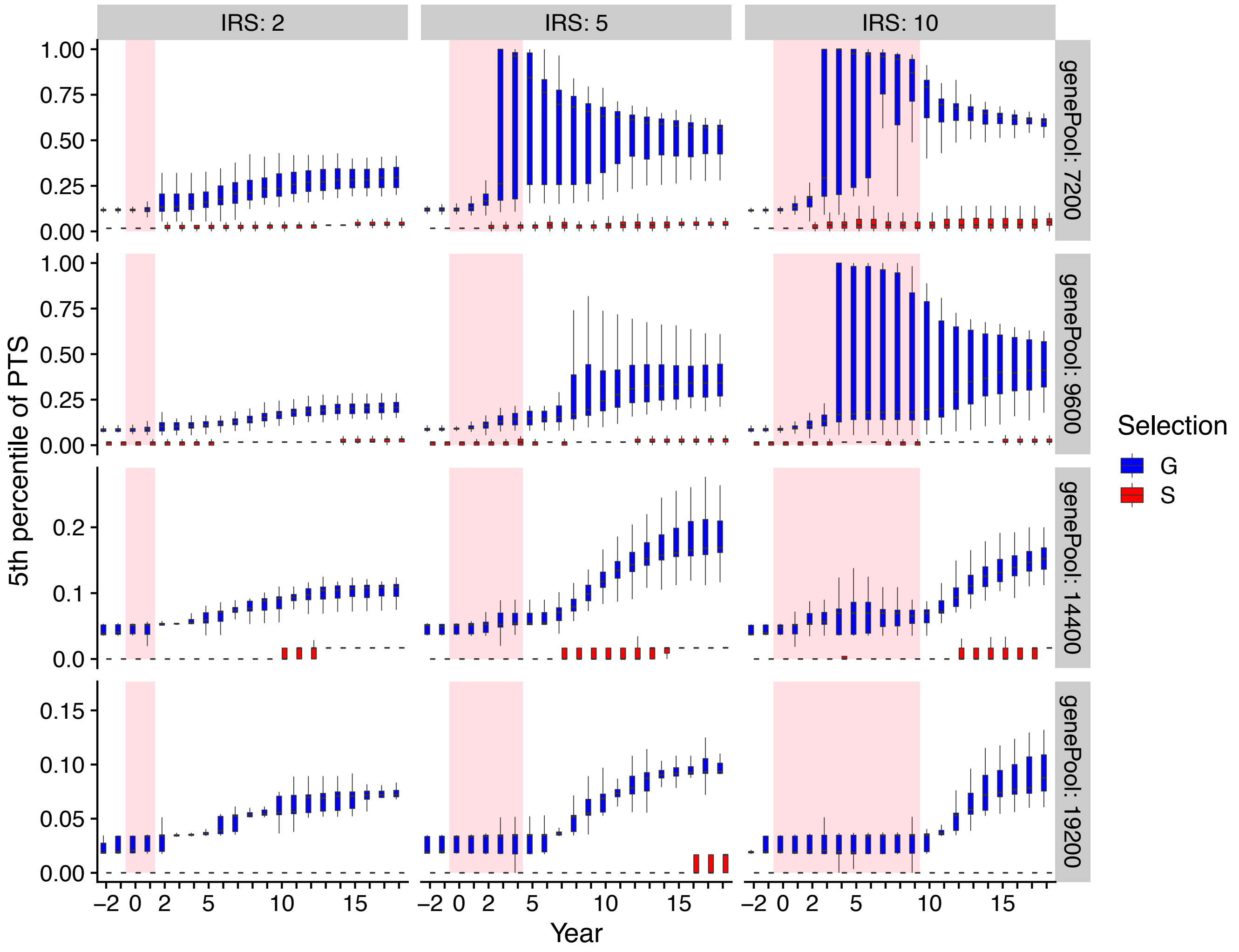

Figure S4. Comparisons of the 5th percentile of PTS over time between between S and G. Box-plots summarize the distribution of PTS in its 5th percentile across different gene pool sizes and among replication runs. Highlighted areas indicate the intervention (IRS) period. Shaded areas indicate the intervention (IRS) period. Negative years correspond to times prior to IRS.

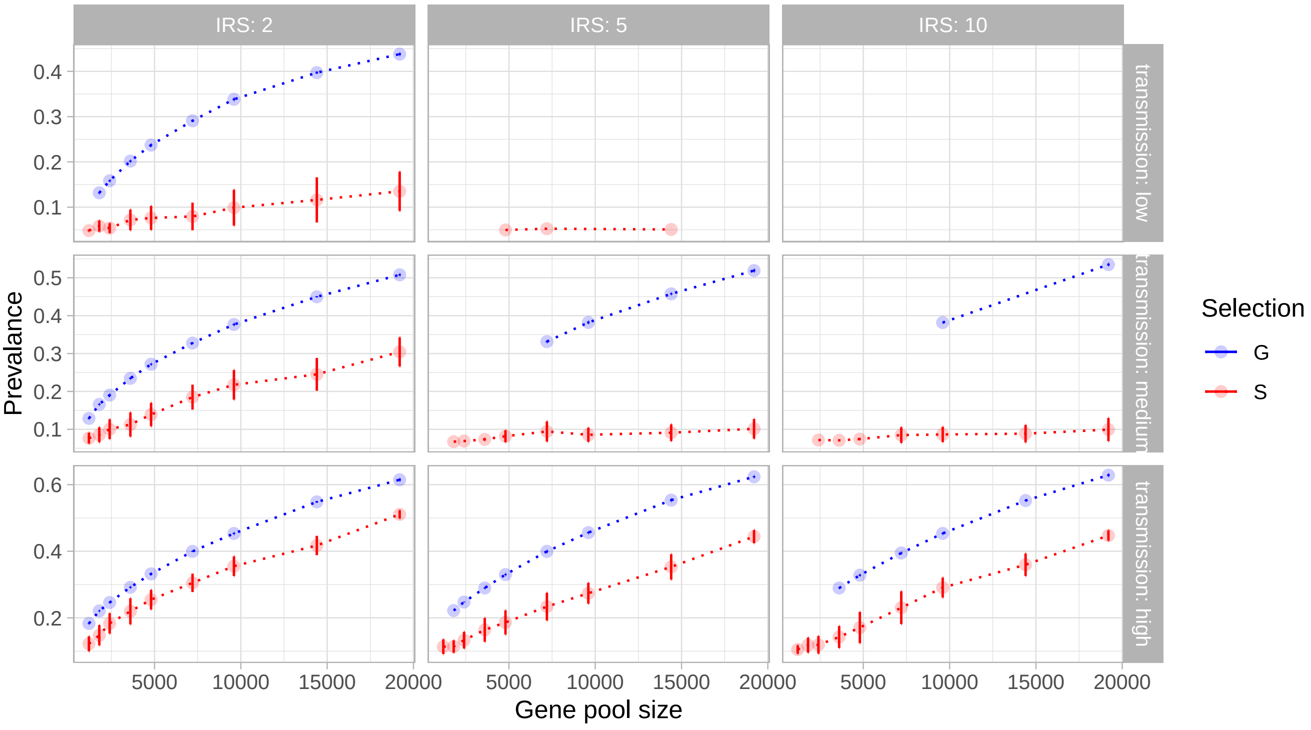

Figure S5. Mean prevalence of surviving runs after IRS as a function of the initial gene pool size given different transmission intensity and length of IRS. Each point shows the mean value (across replications) of the parameter combination with bars representing the standard deviation. Blue color represents simulations under generalized immunity, while red color shows the results from specific immunity.

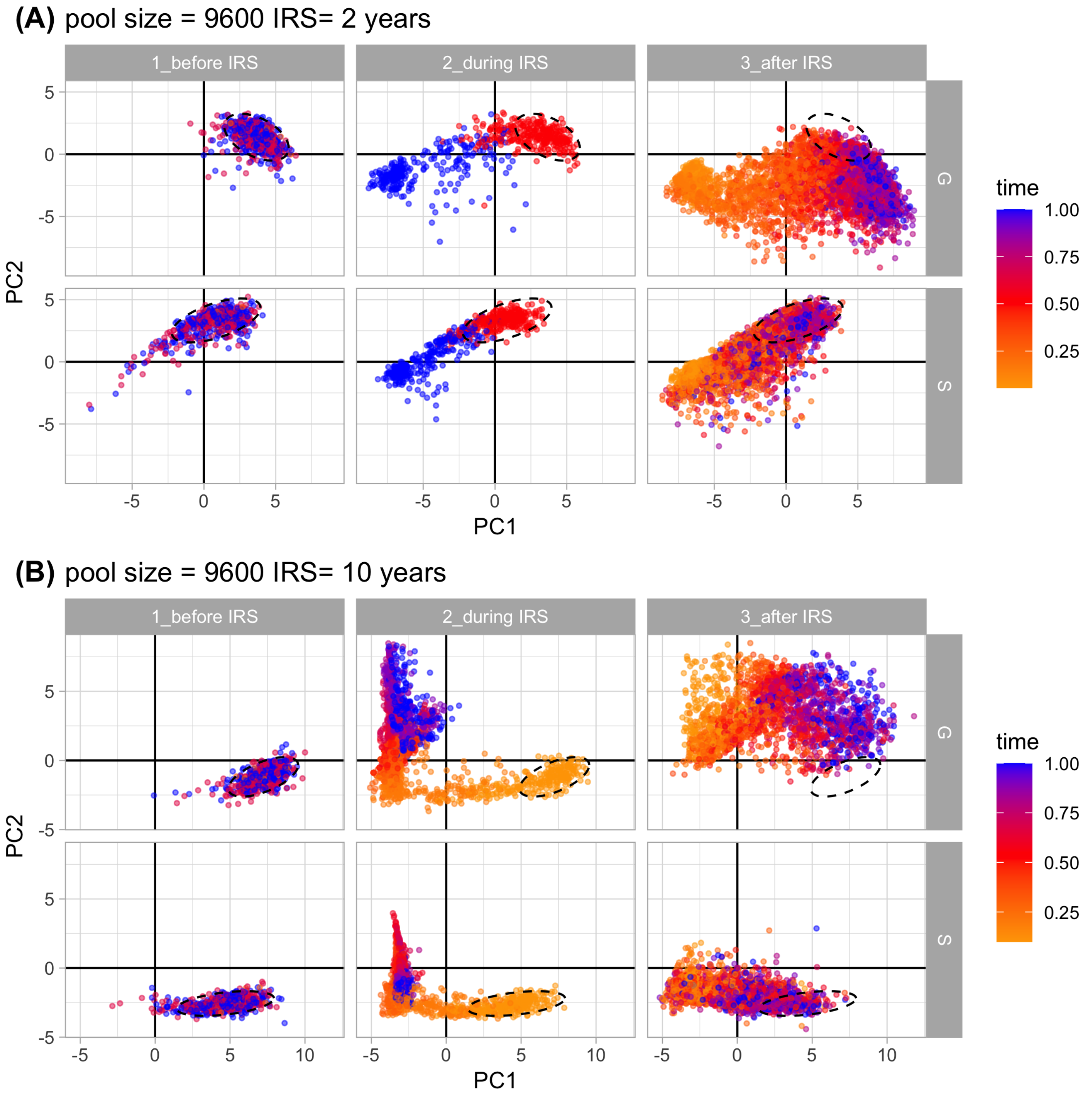

Figure S6. Principal component analysis (PCA) of network properties of *var* repertoire similarity networks.. PCA over time shows how the structure of diversity changes in the 2D space of the PCs for S and G under high transmission. The colors of the networks represent their relative time within the specific time period (i.e., before, during and after IRS) in the simulation. Ellipses show the location of network properties for pre-IRS layers. Parameters: gene pool size equals 9,600 and IRS lasts for 2 and 10 years.

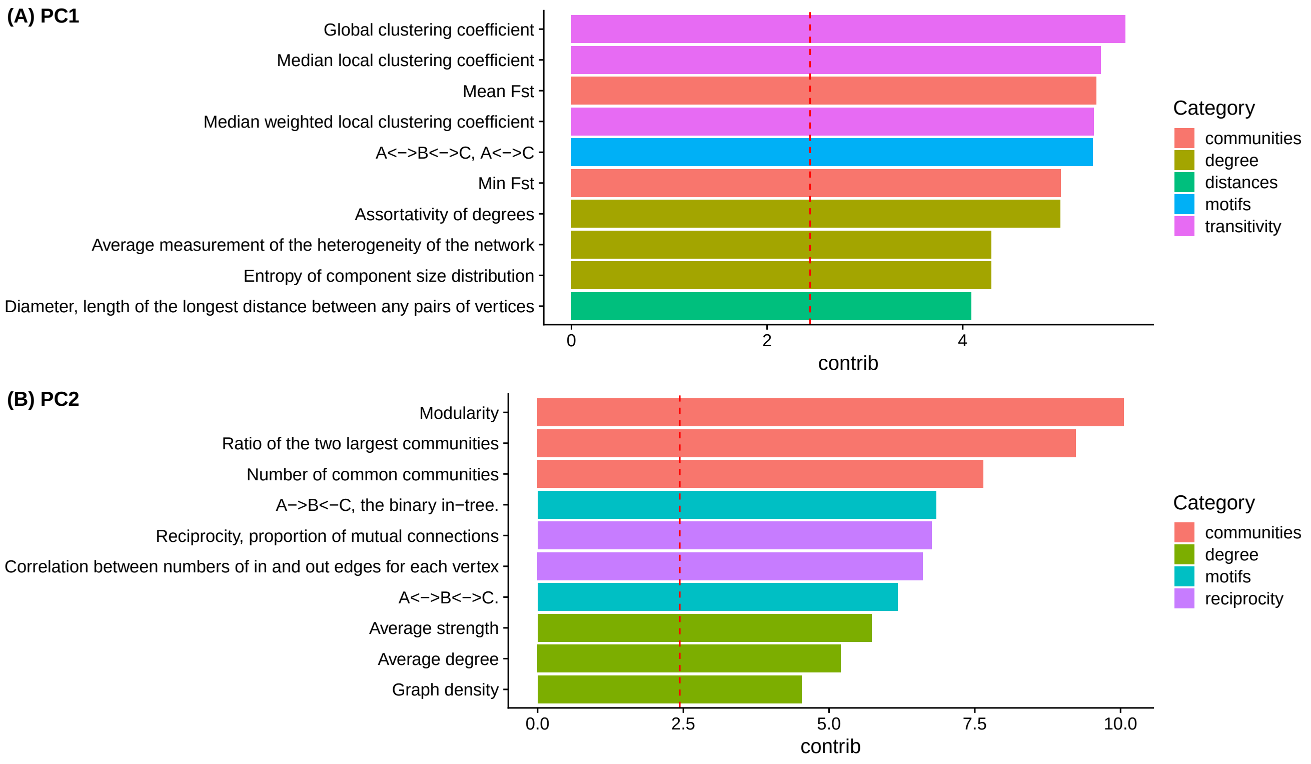

Figure S7. Contributions (in percentage) of network properties to the principal components as shown in Fig. S6. Top 10 properties are shown with a dotted line indicating the expected contribution assuming all variables contribute equally. Color of the bars represents its category of network properties.
